# Medicinal Wines

**Published:** 1879-04

**Authors:** 


MEDICINAL WINES.—We call the attention of physicians to an article of wine imported by Mr. L. Reich, of New York. We can commend the wine imported by Mr. Reich to the medical profession for their purity and medicinal value. It is endorsed by leading men as of value in cases of nervous exhaustion, general debility, and to invalids generally. His goods can only be obtained directly from him, not being on sale in any drug or liquor store.

